# Causality between Alzheimer disease and delirium: A two-sample Mendelian randomization study and gene colocalization analyses

**DOI:** 10.1097/MD.0000000000047492

**Published:** 2026-01-30

**Authors:** Guodong Guo, Hao Ding, Yang Wang, Peng Fang, Xin Wang, Runyang Qian, Huijuan Ma

**Affiliations:** a Department of Orthopedics, Nanjing Jinling Hospital, Affiliated Hospital of Medical School, Nanjing University, Nanjing, China; b Department of Mathematical Sciences, University of Liverpool, Liverpool, United Kingdom; c Department of Neurology, Sir Run Run Hospital, Nanjing Medical University, Nanjing, China.

**Keywords:** Alzheimer disease, delirium, gene colocalization analysis, Mendelian randomization

## Abstract

To evaluate the possible causality between Alzheimer disease (AD) and delirium, a two-sample Mendelian randomization (MR) method and gene colocalization strategy were employed. Genome-wide association study (GWAS) data accessible to the public were utilized. The preliminary findings revealed a causal link between AD (21,982 cases and 41,944 controls) and delirium (comprising 1269 cases and 209,487 controls). By performing MR estimates on an expanded sample, which included AD (90,338 cases and 1036,225 controls) as well as delirium (1269 cases and 209,487 controls), the subsequent analysis confirmed the results. The approaches utilized comprised Inverse-variance weighted (IVW), MR-Egger, weighted median, and weighted mode. To evaluate heterogeneity, Cochran *Q* test was employed, while the MR-Egger intercept and MR-PRESSO tests assessed pleiotropy. Sensitivity was evaluated via leave-one-out analysis. Colocalization results revealed shared genetic loci. The analysis using MR indicated a notable causal influence of AD on delirium occurrence (initial: OR = 1.319, 95% CI: 1.175–1.481, *P* < .001; replication: OR = 1.334, 95% CI: 1.204–1.479, *P* < .001). Meta-analysis confirmed this effect (OR = 1.320, 95% CI: 1.210–1.440, *P* < .01). No heterogeneity or pleiotropy was detected, and results remain robust in sensitivity analyses. Colocalization analysis showed strong shared genetic signals (PPH4 > 0.8), identifying NECTIN2 and TOMM40 as potential mediators. No evidence of reverse causality was identified linking delirium to AD. AD poses a risk for delirium, probably mediated by shared genetic variants NECTIN2 and TOMM40 that impact acetylcholine pathways. These findings highlight potential preventive targets for delirium in Alzheimer patients.

## 1. Introduction

Delirium represents a serious neurological and psychiatric condition marked by notable alterations in consciousness, cognition, and behavior.^[1]^ Patients may present with symptoms such as confusion, disorientation, drowsiness or even coma regarding their level of consciousness.^[2,3]^ Regarding cognition, patients may experience memory loss, inattention, slowed thinking and language impairment. Behavioral symptoms may include anxiety, agitation, violent behavior as well as hallucinations and delusions.^[4]^ Delirium occurs more frequently in older adults, especially in those who experience cognitive decline or have neurological conditions. Estimates suggest that delirium incidence in this age group varies between 10% and 30%.^[3]^ Delirium can result in serious harm to patients, including complications such as malnutrition, dehydration, infection and even death. Additionally, it may cause abnormal behavior such as falls, self-injury or aggression. Consequently, experiencing delirium may result in extended hospital admissions and higher expenses related to healthcare.^[5–9]^ Therefore, timely identification, prevention, and management of delirium are imperative.

Alzheimer disease (AD) is a neurodegenerative condition with deficits in cognition and behavior.^[10]^ The causality between AD and delirium is still a topic of debate. Some studies suggest that AD could potentially increase the risk of delirium, given that those affected by AD often display cognitive and behavioral issues that might lead to the onset and progression of delirium. Furthermore, neuronal death and synaptic damage frequently occur in people diagnosed with AD. These alterations can lead to irregularities in neurotransmitter release, potentially resulting in delirium.^[11,12]^ However, other research efforts indicate that delirium could serve as an initial indicator of AD, potentially resulting in cognitive and behavioral issues that might accelerate AD advancement.^[5,13]^ Elderly patients may exhibit a combination of symptoms indicating AD history, while delirium also tends to manifest prominently within the geriatric population. Therefore, it is essential to investigate the intricate connection between AD and delirium, as this may help mitigate the occurrence of delirium within this particular cohort of elderly patients.

The method of Mendelian randomization (MR), a powerful instrument within the field of epidemiological research, explores the causality between risk factors and particular diseases.^[14]^ This method is grounded in the theory that randomly distributing alleles to offspring mirrors the design of a randomized controlled trial. This methodology effectively mitigates the impact of confounding variables and reverse causation.^[15]^ While commonly applied to examine risk factors, there has yet to be any research utilizing it to explore the causality between AD and delirium. Consequently, to enhance strategies for delirium prevention, MR analysis was utilized to investigate the possible causality linking these 2 conditions.

## 2. Methods

### 2.1. Ethical approval and study design

A comprehensive summary database, Genome-Wide Association Study (GWAS), was used. It is important to note that participants in the original studies provided informed consent, ensuring that ethical standards were upheld throughout the data collection process. Since we depend solely on summary-level data, there is no need for additional ethical approval.

The data from the GWAS employed in the preliminary MR analysis concerning AD was from the International Genomics of Alzheimer Project (IGAP). As part of IGAP, an extensive GWAS meta-analysis was performed, drawing on information from 46 case-control investigations that together encompassed 63,926 individuals of European ancestry. This collection included 21,982 cases of AD and 41,944 normal participants identified as controls.^[16]^ The data can be accessed through the IEU GWAS database (https://gwas.mrcieu.ac.uk/), with the GWAS ID “ieu-b-2.” For the replicate MR analysis concerning AD, the GWAS data were sourced from the PSYCHIATRIC GENOMICS consortium (https://pgc.unc.edu/for-researchers/download-results/), which features a meta-analysis of European participants, consisting of 90,338 cases (46,613 proxy) and 1036,225 controls (318,246 proxy). Finding participants who have late-onset Alzheimer disease (LOAD) is difficult due to the condition typically manifesting later in life. As a result, utilizing proxy cases presents a useful method for incorporating younger subjects into the research by evaluating their risk for LOAD based on the estimated status of their parents. The classification of proxy cases and controls was determined by the established parental LOAD status, with further adjustments made according to the age of the parents.^[17]^

Furthermore, summary statistics regarding delirium, excluding instances triggered by alcohol and various psychoactive substances, were sourced from the FinnGen consortium (round 5, https://r5.finngen.fi/). This dataset comprised 1269 cases alongside 209,487 controls, all of whom are of European descent. Diagnoses of delirium were established using the International Statistical Classification of Diseases (ICD) codes, particularly the ICD-10 code F05. Additionally, the data is accessible via the IEU GWAS database using the GWAS ID “finn-b-F5_DELIRIUM.” Specific information on the relevant data can be found in Table S1 (Supplemental Digital Content, https://links.lww.com/MD/R295).

**Figure 1.**
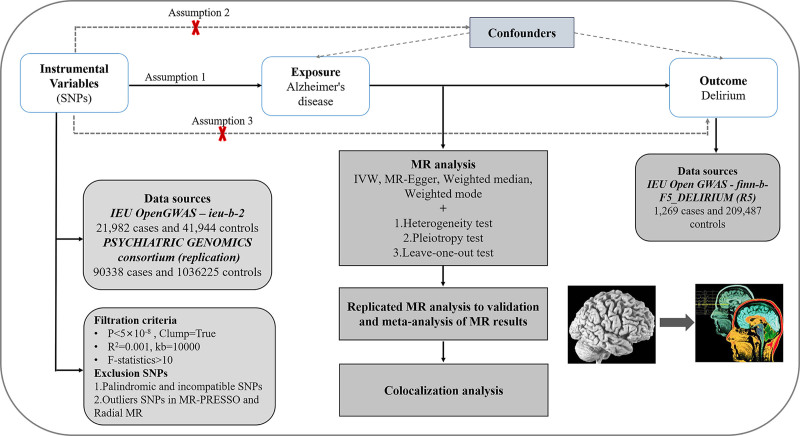
Flowchart of the whole study.

To evaluate the causality linking AD and delirium, the single-nucleotide polymorphisms (SNPs) were used as instrumental variables (IVs). They needed to satisfy 3 key assumptions in order to be valid.^[14,18–21]^ The first assumption states that they should exhibit a strong correlation with AD. The second assumption indicates that they must not be associated with delirium. The third assumption asserts that no variables should confound them. The analytical process comprised 7 essential components: 1) gathering GWAS data related to both AD and delirium; 2) carefully selecting the most appropriate SNPs to serve as IVs for AD; 3) extracting the SNPs of the aforementioned IVs from delirium GWAS data; 4) normalizing the GWAS data for AD and delirium to maintain uniformity; 5) performing MR analysis with assessments of heterogeneity, pleiotropy, and sensitivity; 6) conducting replicated MR analysis and meta-analysis of the MR findings; 7) executing a colocalization analysis. Figure 1 depicts the flowchart representing the complete process.

### 2.2. IVs selection

The exposure was AD, while the outcome was identified as delirium. Genetic variants SNPs with strong correlation (*P* < 5 × 10^−8^) to AD were collected as IVs one.^[14]^ A threshold of 0.001 was established for the linkage disequilibrium (LD) parameter (*r*^2^), along with a genetic distance of 10.000 kb, to enable the identification of independent SNPs and the elimination of LD effects on the findings. The *F*-statistic assessed the strength of the relationship between IVs and exposure factors. To prevent bias from weak IVs, we focused exclusively on SNPs with an *F*-statistic over 10.^[22]^

### 2.3. Statistical analysis

The MR analysis focusing on AD and delirium was performed using the “TwoSampleMR” package in R (version 4.1.2). The random-effects variance-weighted model (IVW) was predominant, with the MR-Egger, weighted median, and weighted mode used as supplements to guarantee robust results.^[18]^ To evaluate the heterogeneity, the I2 index was utilized along with Cochran *Q* and Rucker *Q* statistics, with a *P*-value above .05, suggesting a lack of heterogeneity.^[18]^ Additionally, both the MR-Egger approach and the MR pleiotropy residual sum and outlier (MR-PRESSO) technique were used to examine horizontal pleiotropy. Although the MR-Egger method might demonstrate reduced accuracy in certain situations, the MR-PRESSO technique can detect outlier SNPs and possible horizontal pleiotropy.^[23]^ We eliminated SNPs identified as pleiotropic outliers using MR-PRESSO and Radial MR (*P* < .05).^[18,24]^ After excluding these outliers, the MR results were recalculated to confirm their reliability. A ‘leave one out’ analysis was conducted to determine if any SNP impacts the causality between exposure and outcome. The results showed *P* > .05, suggesting no horizontal pleiotropy.^[25]^ Furthermore, we investigated the shared genetic etiology and causal links between AD and delirium by employing gene colocalization analysis. This statistical evaluation was conducted using the R package “coloc.”^[26]^ Since the data for AD came from 2 separate GWAS datasets, while the data for delirium were from the same GWAS datasets, we ensured that all analyses were modified according to the Bonferroni correction. A *P*-value of <.025 was deemed statistically significant.

## 3. Results

### 3.1. Initial MR analysis results

**Figure 2.**
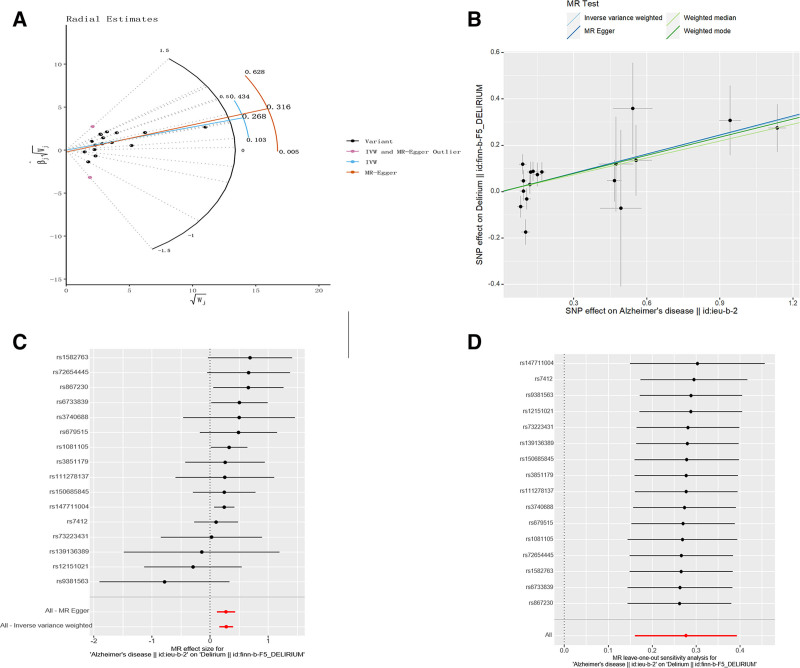
Related results in the initial MR analysis: (A) Outlier SNPs identified by MR Radial. (B) Scatter plot depicting the MR results for AD and delirium. (C) Forest plots illustrating the MR results for AD and delirium. (D) Leave-one-out analysis of the MR results for AD and delirium. AD = Alzheimer disease, MR = Mendelian randomization, SNP = single-nucleotide polymorphisms.

21 SNPs were discovered that exhibit a strong correlation with AD, and subsequently extracted from the delirium GWAS dataset. However, the dataset contained only 20 of the originally identified SNPs. Despite our efforts to identify proxy SNPs that could serve as substitutes for the missing ones, we were unable to make any significant discoveries in this regard. Among these, 2 SNPs (rs11257242 and rs114812713) were excluded during the harmonization process due to their palindromic nature. To further investigate the data, we conducted 2 types of analyses: MR-PRESSO and Radial MR, aimed at identifying any outlier SNPs that could potentially skew our results. The MR-PRESSO analysis did not yield outlier SNPs, whereas 2 outlier SNPs (rs11767557 and rs12590654) were found in the Radial MR analysis (Fig. 2A). We focused on SNPs that exhibited *F*-statistics >10. As a result, our MR analysis ultimately included a total of 16 SNPs. More detailed information regarding the specific SNPs analyzed was in Table S2 (Supplemental Digital Content, https://links.lww.com/MD/R295).

**Figure 3.**
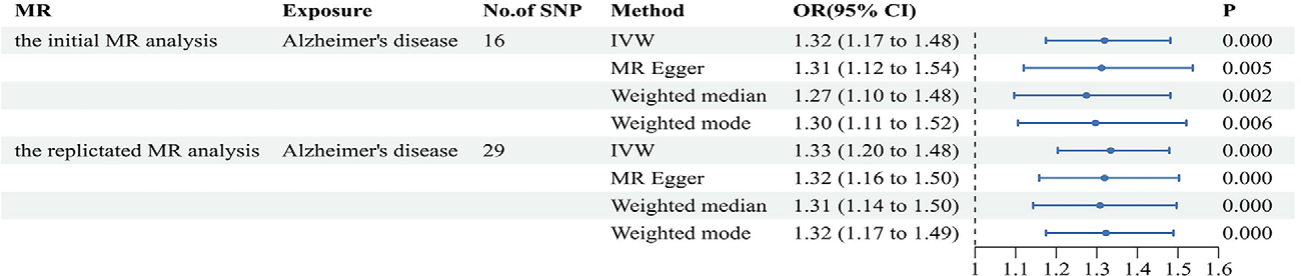
Forest plots of the causal relationship between AD and delirium in the initial MR analysis and the replicated MR analysis. AD = Alzheimer disease, MR = Mendelian randomization.

To assess the causality between AD and delirium, we used IVW as the main analytical approach, with others as supplements. The IVW findings showed a positive association between AD and delirium (OR = 1.319, 95% CI = 1.175–1.481, *P* < .001; Figs. 2B, C, and 3). The Cochran *Q* test revealed no signs of heterogeneity (*Q* value = 12.302, *P *= .582). Additionally, no significant intercept was found (intercept = 0.002, *P *= .921), suggesting no pleiotropy. Evidence of horizontal pleiotropy is absent (RSSobs = 13.377, *P *= .705). Detailed pleiotropy and heterogeneity results can be found in Table 1. The “leave-one-out” results confirmed the stability of MR findings, which were unaffected by any single SNP (Fig. 2D).

**Table 1:** Heterogeneity and pleiotropy tests for AD-delirium MR in initial and replicated analyses.

MR analysis	Exposure	Outcome	Cochran *Q* test (*P*-value, IVW)	Rucker *Q* test (*P*-value, MR-egger)	Egger intercept (*P*-value, MR-egger)	MR-PRESSO global test (*P*-value)
The initial MR analysis	Alzheimer disease	Delirium	.656	.582	.922	.705
The replictated MR analysis	Alzheimer disease	Delirium	.441	.393	.776	.512

AD = Alzheimer disease, IVW = inverse-variance weighted, MR = Mendelian randomization.

### 3.2. The results of the replicated MR analysis

To confirm the reliability of the causal link, a replicated MR analysis was performed. The GWAS dataset related to AD was sourced from 2 European origins. Although we made thorough attempts to gather delirium GWAS data from various sources, we were unsuccessful in this endeavor and consequently relied on the same delirium GWAS dataset for our research. We ensured that all analyses were adjusted using the Bonferroni method. *P* < .025 was considered statistically significant.

**Figure 4.**
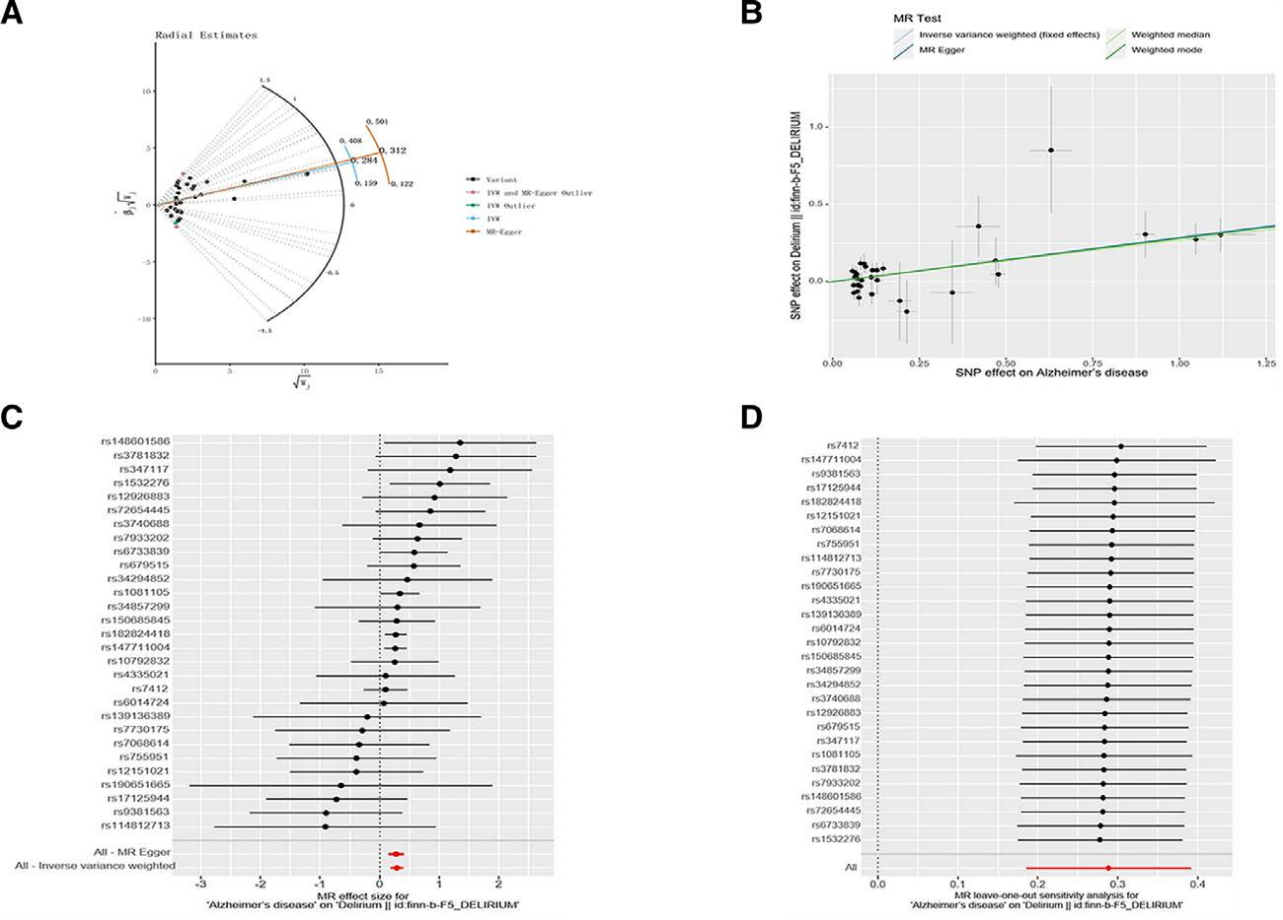
Related results in the replicated MR analysis: (A) Outlier SNPs identified by MR radial. (B) Scatter plot depicting the MR results for AD and delirium. (C) Forest plots illustrating the MR results for AD and delirium. (D) Leave-one-out analysis of the MR results for AD and delirium. AD = Alzheimer disease, MR = Mendelian randomization, SNP = single-nucleotide polymorphisms.

We identified 32 SNPs with a strong correlation to AD, which we extracted from the delirium GWAS dataset. None of these SNPs were removed during the harmonization process due to being palindromic. We performed analyses to pinpoint outlier SNPs. Although the MR-PRESSO analysis revealed no outlier SNPs, the Radial MR analysis identified 3 specific outlier SNPs (rs12590654, rs7584040, and rs9640384) as shown in Figure 4A. As a result, we ultimately incorporated 29 SNPs into the MR analysis. We focused exclusively on SNPs with an *F*-statistic over 10. More detailed information regarding the specific SNPs analyzed was in Table S3 (Supplemental Digital Content, https://links.lww.com/MD/R295).

Our IVW findings similarly revealed a positive relationship between AD and delirium (OR = 1.334, 95% CI = 1.204–1.479, *P* < .001; Figs. 3 and 4B, C). The Cochran *Q* test showed no heterogeneity (*Q* value = 28.353, *P *= .393), and a significant intercept was unobserved (intercept = 0.004, *P *= .776), suggesting absent pleiotropy. Furthermore, evidence of horizontal pleiotropy is absent (RSSobs = 29.230, *P *= .512). The outcomes of the pleiotropy and heterogeneity assessments are summarized (Table 1). Our “leave-one-out” analysis confirmed the stability of the MR findings, which were unaffected by any single SNP (Fig. 4D).

### 3.3. The results of the meta-analysis and colocalization analysis

**Figure 5.**
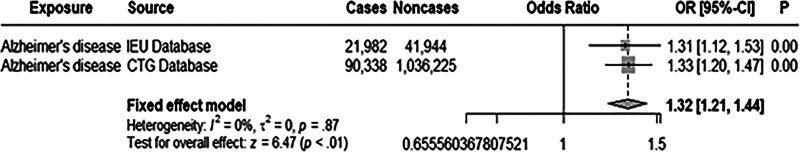
Meta-analysis results of the AD and delirium in the initial and replicated MR analysis. AD = Alzheimer disease, MR = Mendelian randomization.

A meta-analysis examining the initial and replicated MR analyses was conducted, revealing a continued positive association between AD and delirium (OR = 1.320, 95% CI = 1.210–1.440, *P* < .01) (Fig. 5). Reverse MR analysis, with delirium as the exposure and AD as the outcome, was also performed (Tables S4 and S 5, Supplemental Digital Content, https://links.lww.com/MD/R295). This suggests that delirium might not serve as a contributor to AD development.

**Figure 6.**
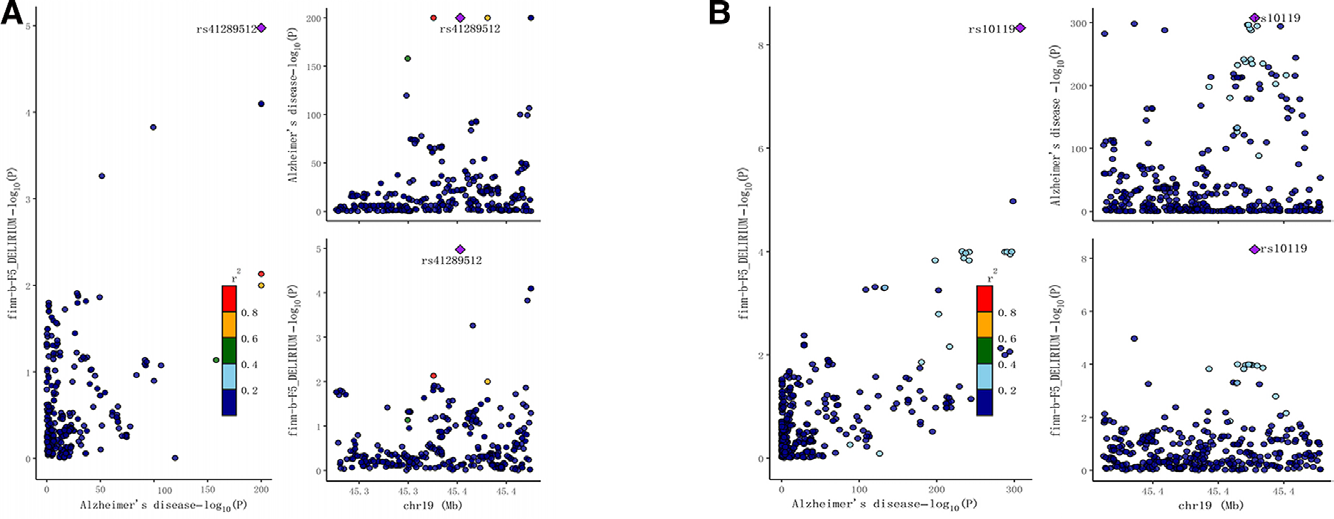
Regional Locus Zoom plots and colocalization analyses results: (A) The results of the AD and delirium in the initial MR analysis. (B) The results of the AD and delirium in the replicated MR analysis. AD = Alzheimer disease, MR = Mendelian randomization.

We employed the coloc R package to conduct a colocalization analysis of GWAS results for AD and delirium.^[26]^ SNPs located within 50KB both upstream and downstream of the leading SNP for AD were chosen, and we then identified the overlap with SNPs associated with delirium.^[27]^ Bayesian algorithms were used to calculate significant colocalization associations in this region.^[28]^ Locus Zoom plots were created to visualize these colocalization result.^[29]^ In the preliminary and replicated MR analyses, we observed strong colocalization between AD and delirium, with posterior probabilities of 97.18% and 99.99%, respectively, for shared signals (Fig. 6).

## 4. Discussion

This research employed MR to investigate the possible causality linking AD and delirium, along with the mechanisms involved, utilizing colocalization analysis. Our findings revealed a notable causal connection between AD and delirium, indicated by an OR of 1.319 (95% CI = 1.175–1.481, *P* < .001). This was successfully validated in our cohort, where we observed OR values of 1.334 (95% CI = 1.204–1.479, *P* < .001). Furthermore, a positive relationship persisted in a meta-analysis, yielding OR values of 1.320 (95% CI = 1.210–1.440, *P* < .01). In the preliminary and replicated MR analyses, we detected strong colocalization between AD and delirium, with posterior probabilities of 97.18% and 99.99%, respectively, for shared signals. These indicated that AD might serve as a risk factor for delirium development.

Our research uncovered a significant connection between AD and delirium, highlighting shared genetic variants. Nevertheless, in the reverse MR analysis, no robust link between delirium and AD was observed. While numerous studies have explored the relationship between these 2 conditions, the causal relationship continues to be a subject of discussion. AD patients typically exhibit cognitive and behavioral abnormalities, which may lead to the occurrence and development of delirium. This is because AD can cause neuronal death and synaptic damage in the brain, which may result in abnormal release of neurotransmitters and thus the occurrence and development of delirium.^[11]^ On the other hand, delirium may be an early symptom of AD, as it may cause cognitive and behavioral abnormalities and accelerate the progression of AD.^[12]^ Additionally, it is suggested that there may not be a direct causal link between AD and delirium. Delirium can be caused by a combination of factors, such as drug side effects, infections, metabolic disorders, inflammatory responses, stress responses, and neurotransmitter imbalance.^[5]^

AD is a neurodegenerative disorder associated with the misfolding and aggregation of proteins, resulting in the formation of senile plaques composed of extracellular Amyloid-β (Aβ) peptides and Intracellular Neurofibrillary Tangles (NFT) composed of hyperphosphorylated tau protein.^[30,31]^ Many studies have demonstrated that the abnormal aggregation of Aβ can impact the synthesis and release of acetylcholine, and may also interfere with acetylcholine receptors on neuronal membranes, ultimately disrupting acetylcholine functio.^[32,33]^ Acetylcholine is a crucial neurotransmitter that plays a role in various cognitive and mood-related functions, including learning, memory, attention, and mood regulation. Impairment of its function can lead to clinical symptoms of cognitive impairment, such as hallucinations and confusion, which are consistent with the symptoms associated with delirium.^[34]^ Although the pathogenesis of delirium is not yet fully understood, neurotransmitter imbalance is believed to be the main cause, with acetylcholine being the neurotransmitter most closely associated with delirium.^[35–37]^ Many observational studies have confirmed that cholinergic impairment can lead to deliri.^[38–40]^

In our colocalization analysis revealed that AD and delirium share the same genetic variant, with SNPs rs41289512 and rs10119, respectively. The SNP rs41289512 influences the expression of NECTIN2, also known as PVRL2, which plays a role in the pathogenesis of AD. The PVRL2 gene may interact with other factors to substantially increase the risk of AD by affecting Aβ metabolis.^[41,42]^ Similarly, the SNP rs10119 influences the expression of TOMM40, which also plays a crucial role in the pathogenesis of AD, particularly in relation to Aβ metabolism.^[42,43]^ Beyond its established role in cell adhesion and immunity, PVRL2 has been implicated in metabolic dysregulation linked to AD. A recent study demonstrates that genetic variants within the PVRL2 gene are not only associated with an increased risk of AD but also with a specific lipid profile – including elevated triglycerides and remnant lipoprotein particles – characteristic of diabetic dyslipidemia.^[44]^ This suggests that PVRL2’s contribution to AD pathogenesis may be mediated through its modulation of systemic lipid metabolism and the subsequent impact on cerebral amyloid-β homeostasis. Concurrently, the pivotal role of TOMM40 in mitochondrial function is well-documented. The findings reinforce its significance, showing that a variable-length polymorphism in TOMM40 is a key predictor for the age of onset of late-onset AD.^[45]^ This polymorphism’s effect is thought to be driven by its influence on TOMM40 expression levels, which in turn affects mitochondrial protein import, energy production, and oxidative stress responses – all critical factors in AD neurodegeneration. Thus, the genetic link we identified between delirium and AD may reflect a compounded pathophysiological burden: one driven by PVRL2’s pleiotropic effects on both the immune and metabolic systems, and the other by TOMM40’s profound impact on mitochondrial health and the timing of AD clinical manifestation. In summary, one hypothesis that may explain the onset of delirium due to AD is that NECTIN2 and TOMM40 affect acetylcholine function by influencing the metabolism of Aβ, which ultimately leads to the onset of deliriu.

This research utilizes an MR method to establish that AD serves as a risk factor for delirium, while also examining the inherent relationship through a comprehensive review of pertinent literature. A significant benefit of this research compared to conventional observational studies is that causal estimates are free from reverse causality and confounding biases. Nonetheless, certain limitations should be noted. One limitation is that the individuals involved were of European descent, which raises questions about the applicability of the results to other demographics and geographical areas. Another limitation is the need to further explore the specific impacts of various exposures, such as age and educational attainment, on the outcomes.

## 5. Conclusion

Using a MR approach, we have identified AD as a risk factor for the development of delirium and its potential contribution to the onset of Delirium. In addition, AD and delirium shared genetic variants rs41289512 and rs10119. Furthermore, NECTIN2 and TOMM40 may serve as crucial mediators in the onset of delirium in AD, and whether intervening in the expression of NECTIN2 and TOMM40 can prevent delirium in Alzheimer patients requires further investigation.

## Author contributions

**Conceptualization:** Guodong Guo, Huijuan Ma.

**Data curation:** Guodong Guo, Yang Wang, Peng Fang, Xin Wang.

**Formal analysis:** Yang Wang, Peng Fang, Xin Wang, Runyang Qian.

**Investigation:** Yang Wang, Xin Wang, Runyang Qian.

**Supervision:** Hao Ding, Huijuan Ma.

**Validation:** Guodong Guo, Peng Fang.

**Visualization:** Hao Ding, Xin Wang, Runyang Qian.

**Writing – original draft:** Guodong Guo.

**Writing – review & editing:** Hao Ding, Huijuan Ma.

## Supplementary Material



## References

[1] Mattison MLP. Delirium. Ann Intern Med. 2020;173:ITC49–64. 33017552 10.7326/AITC202010060

[2] American Psychiatric Association D-TF. Diagnostic and statistical manual of mental disorders: DSM-5™. 5th ed. American Psychiatric Publishing; 2013.

[3] Naeije G Pepersack T. Delirium in elderly people. Lancet. 2014;383:2044–5. 24931688 10.1016/S0140-6736(14)60993-4

[4] Burton JK Craig LE Yong SQ. Non-pharmacological interventions for preventing delirium in hospitalised non-ICU patients. Cochrane Database Syst Rev. 2021;7:CD013307. 34280303 10.1002/14651858.CD013307.pub2 PMC8407051

[5] Bellelli G Morandi A Davis DH. Validation of the 4AT, a new instrument for rapid delirium screening: a study in 234 hospitalised older people. Age Ageing. 2014;43:496–502. 24590568 10.1093/ageing/afu021 PMC4066613

[6] Bellelli G Morandi A Davis DH. Corrigendum to ‘Validation of the 4AT, a new instrument for rapid delirium screening: a study in 234 hospitalised older people’. Age Ageing. 2015;44:175. 25477307 10.1093/ageing/afu181 PMC4989361

[7] Geriatric Medicine Research C. Improving delirium screening and recognition in UK hospitals: results of a multi-centre quality improvement project. Age Ageing. 2022;51:1–13. 10.1093/ageing/afab243 PMC8876302 35212730

[8] Lozano-Vicario L Garcia-Hermoso A Cedeno-Veloz BA. Biomarkers of delirium risk in older adults: a systematic review and meta-analysis. Front Aging Neurosci. 2023;15:1174644. 37251808 10.3389/fnagi.2023.1174644 PMC10213257

[9] Siddiqi N House AO Holmes JD. Occurrence and outcome of delirium in medical in-patients: a systematic literature review. Age Ageing. 2006;35:350–64. 16648149 10.1093/ageing/afl005

[10] Hshieh TT Fong TG Schmitt EM; BASIL Study Group. Does alzheimer’s disease and related dementias modify delirium severity and hospital outcomes? J Am Geriatr Soc. 2020;68:1722–30. 32255521 10.1111/jgs.16420 PMC7725352

[11] Davis DHJ Skelly DT Murray C. Worsening cognitive impairment and neurodegenerative pathology progressively increase risk for delirium. Am J Geriatr Psychiatry. 2015;23:403–15. 25239680 10.1016/j.jagp.2014.08.005 PMC4278840

[12] Henjum K Quist-Paulsen E Zetterberg H Blennow K Nilsson LNG Watne LO. CSF sTREM2 in delirium-relation to Alzheimer’s disease CSF biomarkers Abeta42, t-tau and p-tau. J Neuroinflammation. 2018;15:304. 30390679 10.1186/s12974-018-1331-1 PMC6215363

[13] Fong TG Vasunilashorn SM Libermann T Marcantonio ER Inouye SK. Delirium and Alzheimer disease: a proposed model for shared pathophysiology. Int J Geriatr Psychiatry. 2019;34:781–9. 30773695 10.1002/gps.5088 PMC6830540

[14] Burgess S Thompson SG. Multivariable Mendelian randomization: the use of pleiotropic genetic variants to estimate causal effects. Am J Epidemiol. 2015;181:251–60. 25632051 10.1093/aje/kwu283 PMC4325677

[15] Walker VM Zheng J Gaunt TR Smith GD. Phenotypic causal inference using genome-wide association study data: mendelian randomization and beyond. Annu Rev Biomed Data Sci. 2022;5:1–17. 35363507 10.1146/annurev-biodatasci-122120-024910 PMC7614231

[16] Kunkle BW Grenier-Boley B Sims R; Alzheimer Disease Genetics Consortium (ADGC). Genetic meta-analysis of diagnosed Alzheimer’s disease identifies new risk loci and implicates Abeta, tau, immunity and lipid processing. Nat Genet. 2019;51:414–30. 30820047 10.1038/s41588-019-0358-2 PMC6463297

[17] Wightman DP Jansen IE Savage JE; 23andMe Research Team. A genome-wide association study with 1,126,563 individuals identifies new risk loci for Alzheimer’s disease. Nat Genet. 2021;53:1276–82. 34493870 10.1038/s41588-021-00921-z PMC10243600

[18] Bowden J Spiller W Del Greco MF. Improving the visualization, interpretation and analysis of two-sample summary data Mendelian randomization via the Radial plot and Radial regression. Int J Epidemiol. 2018;47:1264–78. 29961852 10.1093/ije/dyy101 PMC6124632

[19] Lawlor DA Harbord RM Sterne JA Timpson N Smith GD. Mendelian randomization: using genes as instruments for making causal inferences in epidemiology. Stat Med. 2008;27:1133–63. 17886233 10.1002/sim.3034

[20] Smith GD Ebrahim S. ‘Mendelian randomization’: can genetic epidemiology contribute to understanding environmental determinants of disease? Int J Epidemiol. 2003;32:1–22. 12689998 10.1093/ije/dyg070

[21] Smith GD Ebrahim S. Mendelian randomization: prospects, potentials, and limitations. Int J Epidemiol. 2004;33:30–42. 15075143 10.1093/ije/dyh132

[22] Burgess S Thompson SG; CRP CHD Genetics Collaboration. Avoiding bias from weak instruments in Mendelian randomization studies. Int J Epidemiol. 2011;40:755–64. 21414999 10.1093/ije/dyr036

[23] Verbanck M Chen CY Neale B Do R. Detection of widespread horizontal pleiotropy in causal relationships inferred from Mendelian randomization between complex traits and diseases. Nat Genet. 2018;50:693–8. 29686387 10.1038/s41588-018-0099-7 PMC6083837

[24] Ong JS MacGregor S. Implementing MR-PRESSO and GCTA-GSMR for pleiotropy assessment in Mendelian randomization studies from a practitioner’s perspective. Genet Epidemiol. 2019;43:609–16. 31045282 10.1002/gepi.22207 PMC6767464

[25] Hemani G Bowden J Smith GD. Evaluating the potential role of pleiotropy in Mendelian randomization studies. Hum Mol Genet. 2018;27:R195–208. 29771313 10.1093/hmg/ddy163 PMC6061876

[26] Wallace C. Eliciting priors and relaxing the single causal variant assumption in colocalisation analyses. PLoS Genet. 2020;16:e1008720. 32310995 10.1371/journal.pgen.1008720 PMC7192519

[27] Giambartolomei C Vukcevic D Schadt EE. Bayesian test for colocalisation between pairs of genetic association studies using summary statistics. PLoS Genet. 2014;10:e1004383. 24830394 10.1371/journal.pgen.1004383 PMC4022491

[28] Venkateswaran S Prince J Cutler DJ. Enhanced contribution of HLA in pediatric onset ulcerative colitis. Inflamm Bowel Dis. 2018;24:829–38. 29562276 10.1093/ibd/izx084 PMC6350448

[29] Pruim RJ Welch RP Sanna S. LocusZoom: regional visualization of genome-wide association scan results. Bioinformatics. 2010;26:2336–7. 20634204 10.1093/bioinformatics/btq419 PMC2935401

[30] Sinha S. The role of beta-amyloid in Alzheimer’s disease. Med Clin North Am. 2002;86:629–39. 12168562 10.1016/s0025-7125(02)00022-6

[31] Breijyeh Z Karaman R. Comprehensive review on Alzheimer’s disease: causes and treatment. Molecules. 2020;25:5789. 33302541 10.3390/molecules25245789 PMC7764106

[32] Madhu P Mukhopadhyay S. Distinct types of amyloid-beta oligomers displaying diverse neurotoxicity mechanisms in Alzheimer’s disease. J Cell Biochem. 2021;122:1594–608. 34494298 10.1002/jcb.30141

[33] Roberts JP Stokoe SA Sathler MF Nichols RA Kim S. Selective coactivation of alpha7- and alpha4beta2-nicotinic acetylcholine receptors reverses beta-amyloid-induced synaptic dysfunction. J Biol Chem. 2021;296:100402. 33571523 10.1016/j.jbc.2021.100402 PMC7961090

[34] Wang Y Shen X. Postoperative delirium in the elderly: the potential neuropathogenesis. Aging Clin Exp Res. 2018;30:1287–95. 30051417 10.1007/s40520-018-1008-8

[35] Hshieh TT Fong TG Marcantonio ER Inouye SK. Cholinergic deficiency hypothesis in delirium: a synthesis of current evidence. J Gerontol A Biol Sci Med Sci. 2008;63:764–72. 18693233 10.1093/gerona/63.7.764 PMC2917793

[36] Li H Han S Feng J. Delirium after deep brain stimulation in Parkinson’s Disease. Parkinsons Dis. 2021;2021:8885386. 33604017 10.1155/2021/8885386 PMC7872740

[37] Rump K Holtkamp C Bergmann L. Midazolam impacts acetyl-And butyrylcholinesterase genes: an epigenetic explanation for postoperative delirium? PLoS One. 2022;17:e0271119. 35802656 10.1371/journal.pone.0271119 PMC9269431

[38] Ah YM Suh Y Jun K Hwang S Lee JY. Effect of anticholinergic burden on treatment modification, delirium and mortality in newly diagnosed dementia patients starting a cholinesterase inhibitor: a population-based study. Basic Clin Pharmacol Toxicol. 2019;124:741–8. 30511428 10.1111/bcpt.13184

[39] Pasina L Colzani L Cortesi L. Relation between delirium and anticholinergic drug burden in a cohort of hospitalized older patients: an observational study. Drugs Aging. 2019;36:85–91. 30484239 10.1007/s40266-018-0612-9

[40] Pasina L Rizzi B Nobili A Recchia A. Anticholinergic load and delirium in end-of-life patients. Eur J Clin Pharmacol. 2021;77:1419–24. 33733683 10.1007/s00228-021-03125-w

[41] Johansen M Joensen S Restorff M. Polygenic risk of Alzheimer’s disease in the faroe islands. Eur J Neurol. 2022;29:2192–200. 35384166 10.1111/ene.15351

[42] Liang X Liu C Liu K. Association and interaction of TOMM40 and PVRL2 with plasma amyloid-beta and Alzheimer’s disease among Chinese older adults: a population-based study. Neurobiol Aging. 2022;113:143–51. 35093267 10.1016/j.neurobiolaging.2021.12.013

[43] Zhu Z Yang Y Xiao Z. TOMM40 and APOE variants synergistically increase the risk of Alzheimer’s disease in a Chinese population. Aging Clin Exp Res. 2021;33:1667–75. 32725468 10.1007/s40520-020-01661-6

[44] Guardiola M Muntané G Martínez I. Metabolic overlap between Alzheimer’s disease and metabolic syndrome identifies the PVRL2 gene as a new modulator of diabetic dyslipidemia. Int J Mol Sci. 2023;24:7415. 37108578 10.3390/ijms24087415 PMC10139078

[45] Roses AD Lutz MW Amrine-Madsen H. A TOMM40 variable-length polymorphism predicts the age of late-onset Alzheimer’s disease. Pharmacogenomics J. 2010;10:375–84. 20029386 10.1038/tpj.2009.69 PMC2946560

